# Well-differentiated cerebellum in an ovarian mature cystic teratoma: a case report and review of the literature

**DOI:** 10.1186/s13256-022-03444-1

**Published:** 2022-06-01

**Authors:** Ahmed AlEssa, Hassan H. AlAhmadi, Ayesha Ahmed, Abdulatif AlMousa, Nasreen G. Hamadeh, Yasir A. Hassan Farah

**Affiliations:** 1 Department of Pathology, College of Medicine, King Fahd Hospital of the University, Imam Abdulrahman Bin Faisal University, Post Box No. 1982, Dammam, 31441 Saudi Arabia; 2 Department of Obstetrics and Gynaecology, College of Medicine, King Fahd Hospital of the University, Imam Abdulrahman Bin Faisal University, Post Box No. 1982, Dammam, 31441 Saudi Arabia; 3 Department of Radiology, College of Medicine, King Fahd Hospital of the University, Imam Abdulrahman Bin Faisal University, Post Box No. 1982, Dammam, 31441 Saudi Arabia

**Keywords:** Cerebellum, Teratoma, Ovary, Cystectomy, Case report

## Abstract

**Background:**

Mature teratoma is the most common germ cell tumor as it represents 95% of germ cell tumors. Although common in children and young adults, ovarian teratoma can occur at any age. Mature teratomas are composed of mature tissues representing elements derived from more than one embryonic germ layer (ectoderm, mesoderm, and endoderm), with ectodermal derivatives being the usual predominant component; however, the finding of a well-differentiated cerebellum is extremely rare.

**Case presentation:**

A 20-year-old Saudi female presented to the emergency department with severe abdominal pain of 1-day duration. Pelvic ultrasound showed a large ovoid- to bilobed-shaped cystic pelvic structure extending to the lower abdomen. The patient underwent left ovarian cystectomy. Microscopic examination showed a cyst wall with skin tissue, including adnexal structures (sebaceous glands), a well-differentiated cerebellum, and mature glial tissue. After extensive sampling, no immature component was identified. Thus, the final diagnosis of a mature cystic teratoma with well-differentiated cerebellum was established. The patient was well postoperatively and was discharged in a stable condition.

**Conclusion:**

We report this case of well-differentiated cerebellum within ovarian teratoma to expand the pool of cases reported in literature of this extremely rare entity, as only 22 cases with such findings have been reported in literature to the best of our knowledge. This finding poses a diagnostic challenge to the pathologist due to its rarity and its similarity to immature teratoma. We thus emphasize that thorough sampling of ovarian teratoma is of paramount importance and to keep the aforementioned diagnosis in mind and not confuse it with immature elements, especially in intraoperative consultation and frozen sections.

## Introduction

Mature teratoma is the most common GCT, represents 95% of GCTs and 30% of ovarian tumors, making it the most common ovarian neoplasm in children and teenagers [1, 2]. Although common in children and young adults, ovarian teratoma can occur at any age [2]. Ovarian torsion is the most frequently encountered complication, but most patients are asymptomatic [3, 4]. Mature teratomas are composed of mature tissues representing elements derived from more than one embryonic germ layer (ectoderm, mesoderm, and endoderm), with ectodermal derivatives being the usual predominant component [5, 6]. Such elements may be from the ectoderm (epidermis, sweat glands, hair, and neural tissue), mesoderm (muscles, adipose tissue, cartilage, and bone) or endoderm (respiratory/gastrointestinal tracts and liver) [5, 6].

As mentioned above, mature cystic ovarian teratoma (MCT) can have different histological elements [5, 6]; however, the finding of a well-differentiated cerebellum is extremely rare. The first case reported of a well-differentiated cerebellum within mature teratoma was by Askanazy in 1907 [7]. To the best of our knowledge, only 22 cases with such findings have been reported. Here, we report a case of ovarian cystic teratoma with a well-differentiated cerebellum in a 20-year-old patient.

## Case presentation

**Fig. 1:**
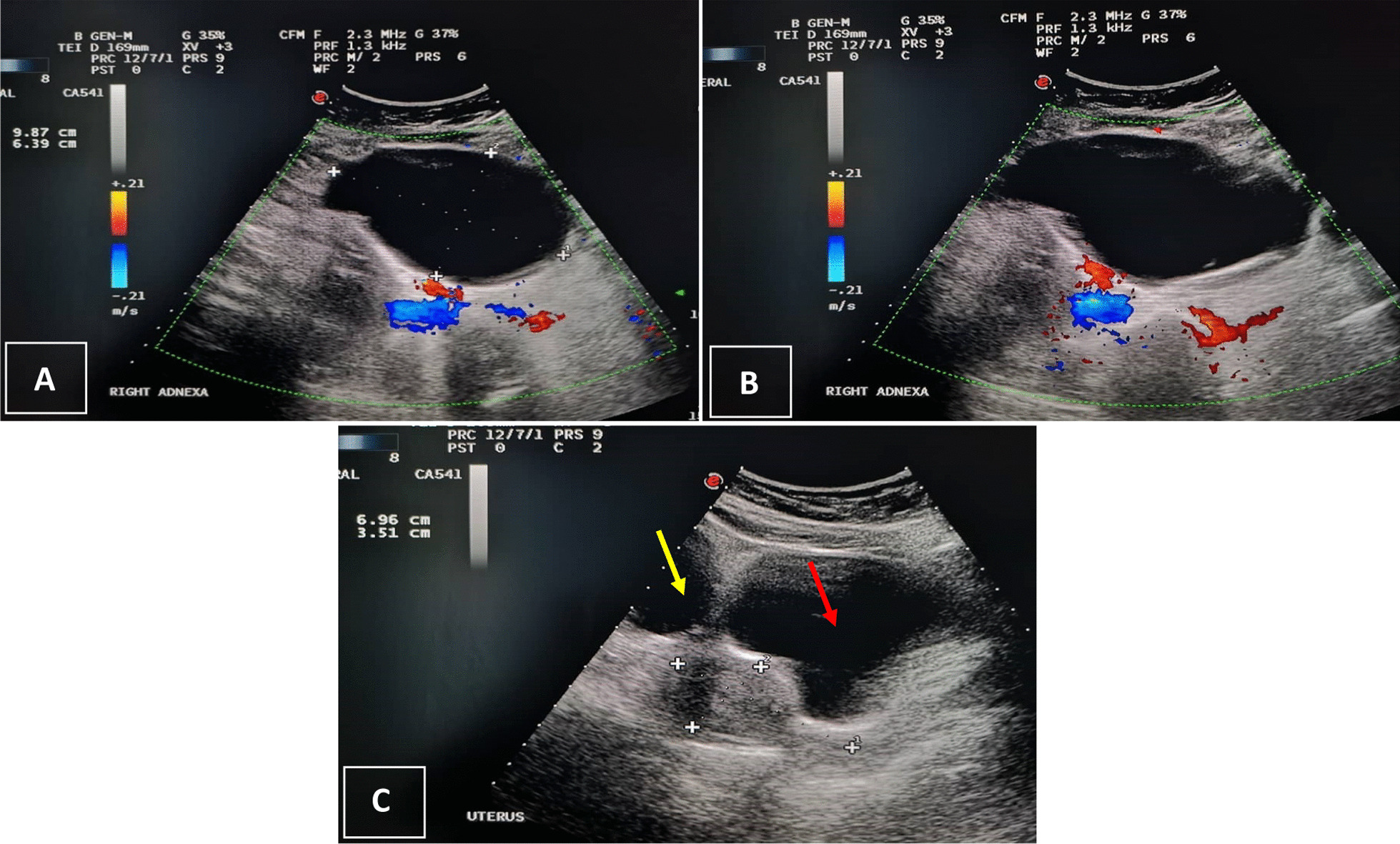
Pelvic ultrasound **A** and **B** showing median and right paramedian pelviabdominal cystic, thin-walled lesion of a pear to ovoid shape with few very thin septa and some hyperechoic material in its dependent part. The lesion shows no calcifications or vascularity internally in its wall. **C** Sagittal/vertical midline view of the pelvis showing the uterus **(++++)** and the urinary bladder (red arrow) just anterior to it, and showing part of the cystic lesion (yellow arrow) located anterosuperior to the uterus

**Fig. 2:**
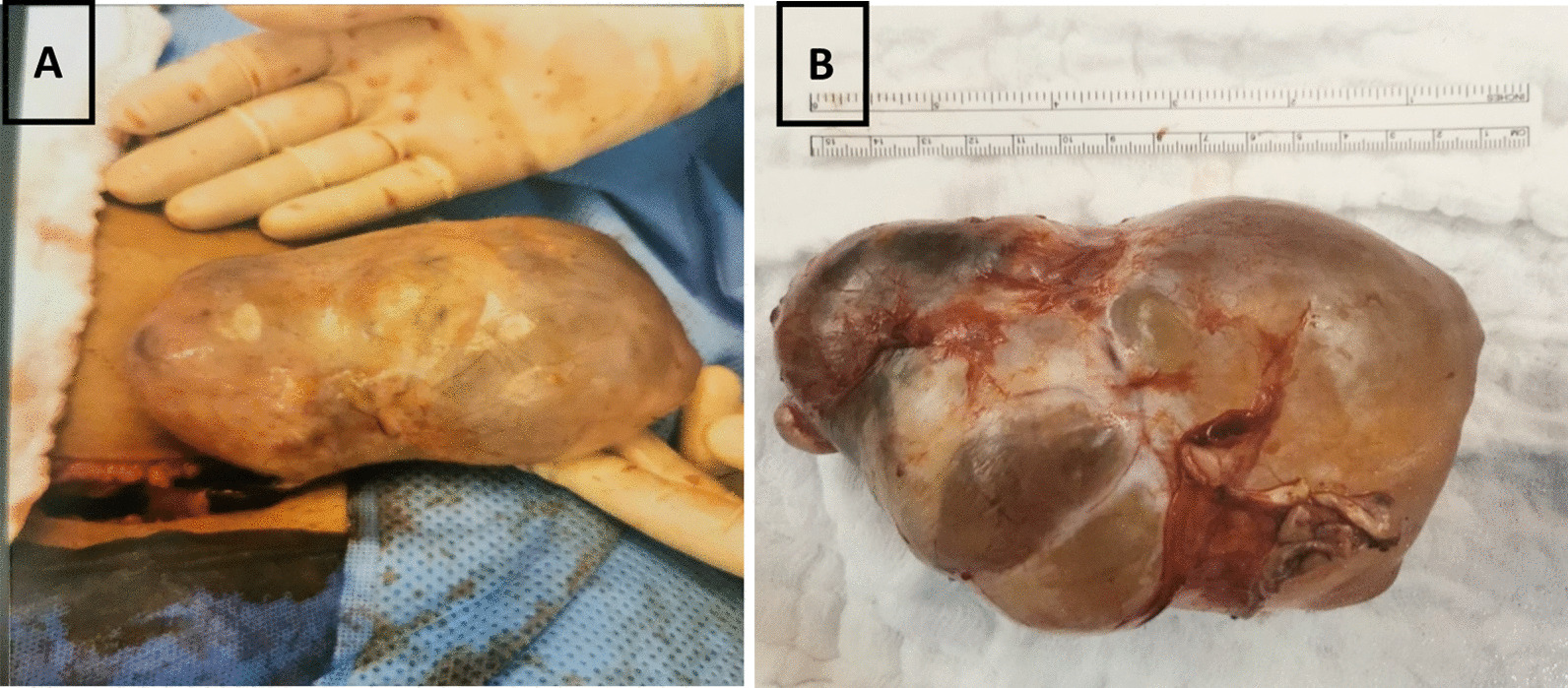
**A**, **B** Intraoperative pictures of the ovarian teratoma

A 20-year-old Saudi female, not known to have any medical illness, presented to the emergency department with history of abdominal pain for 1-day duration. The pain was radiating to the left medial thigh. It was mild at first but then increased in severity, which forced the patient to seek medical care. There were no aggravating or relieving factors, and the pain was not relieved with analgesics. The patient denied any history of similar attacks before. There was no history of other gastrointestinal or urinary symptoms, and no history of per vaginal bleeding. The patient is unmarried, and her menstruation was normal, regular each month lasting around 7 days with normal flow and infrequent mild pain not requiring analgesia. Physical examination showed that the patient was pale and in pain. She was vitally stable. Her laboratory investigations were unremarkable except for microcytic hypochromic anemia with hemoglobin level of 9.9 g/dl. Abdominal examination revealed a soft abdomen with tenderness and mass in the left iliac fossa. Pelvic ultrasound (Fig. 1) showed a large ovoid- to bilobed-shaped cystic pelvic structure extending to the lower abdomen. The cyst measured 12 × 7 cm^2^ with no evidence of calcification. No internal or significant wall vascularity was noted on color Doppler ultrasound. The large cyst was associated with minimal free pelvic fluid, which caused both ovaries to be not visualized. The uterus was of normal size and contexture, with no definite focal lesion or endometrial intraluminal collection. In the context of the pelvic ultrasound findings, which could not rule out ovarian torsion, and the patient’s clinical condition, a decision was taken to proceed to unilateral ovarian cystectomy. Thus, the patient underwent surgery a few hours after admission to the hospital. Intraoperative findings showed a large cyst adherent to the left ovary measuring 15 × 10 cm^2^ with a clear fluid, a solid component, and smooth surface separated from the left ovary. Both the right ovary and the uterus were normal. The specimen was sent intact to the histopathology laboratory in neutral-buffered 10% formalin. Macroscopic examination showed an intact cyst measuring 12 × 8.5 × 5.8 cm^3^. The outer surface was smooth and nodular. On opening, a multilocular cyst filled with clear fluid was identified. The wall of the cyst was thin, measuring 0.1 cm in maximum dimension. Inside the cyst, a soft to firm solid component was identified, tan-yellow in color and measuring 7 × 5 × 2.3 cm^3^, admixed with hair, fat, and cheesy material. The ovary and fallopian tube were not identified. The specimen was extensively sampled and submitted for histopathological examination (Fig. 2).

**Fig. 3:**
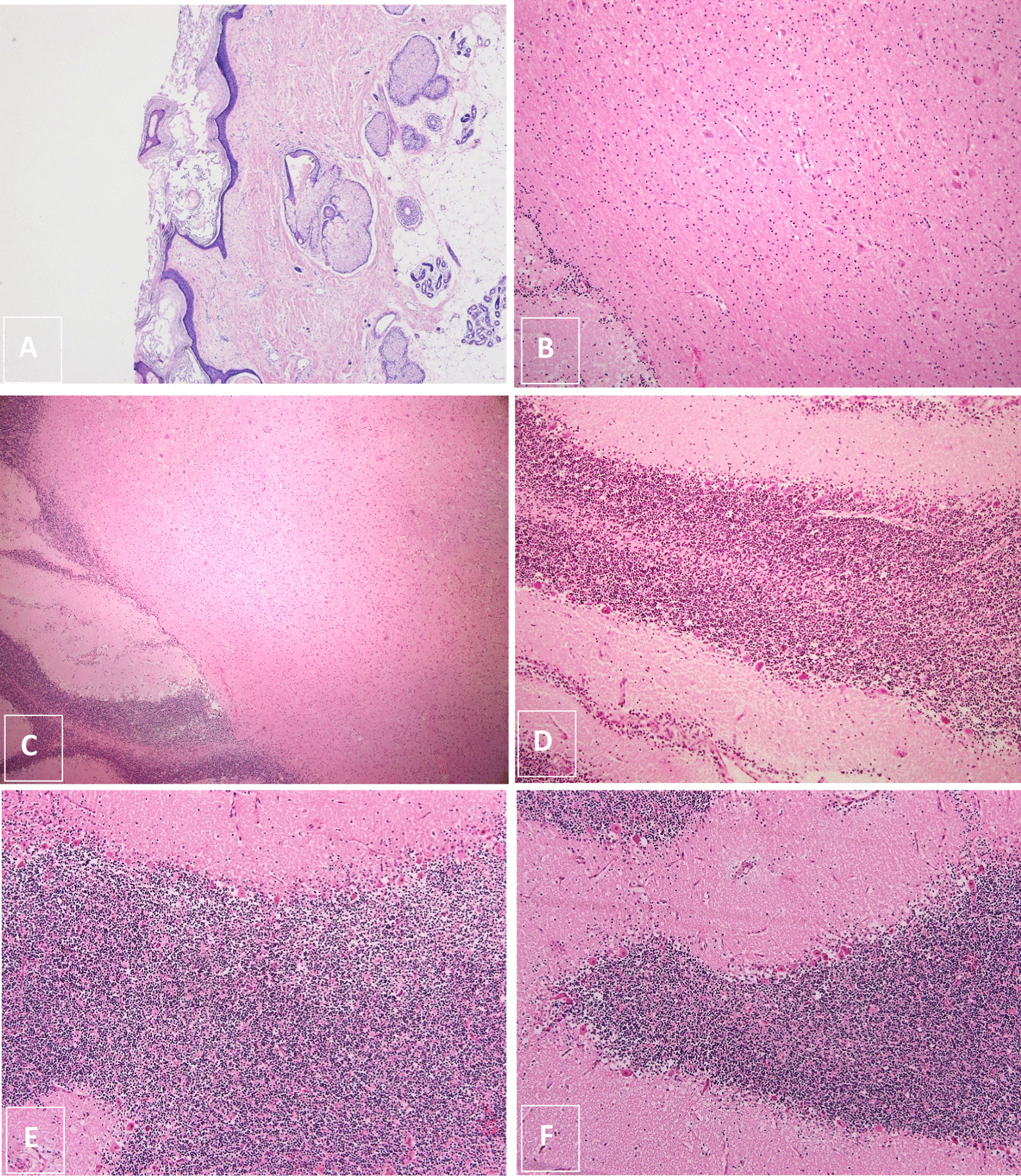
**A** Hematoxylin and eosin-stained sections showing skin along with skin adnexa. **B** Mature glial tissue and neurons. **C** Glial tissue along with part of a cerebellar folium seen on the left. **D–F** Showing mature cerebellum with the outer hypocellular molecular layer, Purkinje cell layer, and inner hypercellular granular layer. (Original magnifications ×40 [**A**, **C**], ×100 [**B**], and ×200 [**D**–**F**])

Microscopic examination of the hematoxylin and eosin (H&E)-stained sections (Fig. 3) showed a cyst wall lined with skin tissue, including adnexal structures (sebaceous glands) and glial tissue, along with a focal area lined by respiratory epithelium. No immature component was identified after extensive sampling. Interestingly, a well-differentiated cerebellar tissue is also seen, composed of an outer hypocellular molecular layer, Purkinje cell layer, and inner hypercellular granular cell layer (Fig. 3).

After extensively sampling the specimen and ruling out the presence of immature elements or a mixed germ cell tumor component, a final diagnosis of a mature cystic teratoma with well-differentiated cerebellum was established based on the above-mentioned findings. Postoperatively, the patient was doing well and was discharged in a stable condition.

## Discussion

Mature teratoma is the most common GCT, as it represents 95% of GCTs and 30% of ovarian tumors, making it the most common ovarian neoplasm in children and teenagers [1, 2]. Although common in children and young adults, ovarian teratoma can occur at any age [2]. It is commonly unilateral but can be bilateral in 9.52% [4] and 10.5% of cases [3]. Ovarian torsion is the most frequently encountered complication; however, most patients are asymptomatic [3, 4, 7]. Other less frequent complications include infection and rupture [8]. Ultrasound is considered one of the most supportive investigation tools as it is safe and readily applied, especially when the patient presents with an acute abdomen, as in our case [9].

Teratoma is classified into mature and immature teratoma [5]. Immature teratoma is defined as having immature elements from any of the three germ layers, commonly with neural origin, but for grading, only the neuroectodermal tissue is considered [5, 10]. Immature teratoma is the most common malignant ovarian germ cell tumor (MOGCT), and its histological grading plays an integral role in the management and prognosis of the patient. Therefore, extensive sampling of the solid areas in teratoma specimens is crucial, which could reveal another germ cell tumor or an immature component [11, 12]. In the presented case, after sampling the specimen thoroughly, only mature tissue elements composed of skin and adnexa, well-differentiated cerebellum, respiratory epithelium, and glial tissue were seen. No evidence of immature elements or mixed germ cell tumor components were identified. Thus, the diagnosis of mature cystic teratoma was established.

Another point to mention is that malignant transformation (MT) is rare in MCT at a rate of 1–2% [13], and various malignancies have been reported in teratomas, with squamous cell carcinoma (SCC) being the most common [13, 14]. However, other reported malignancies include apocrine adenocarcinoma [15] and carcinosarcoma [16]. Treatment of MCT is by surgical removal of the cyst by laparoscopy, with laparotomy reserved for large-sized tumors, as in our case [17, 18]. Recurrence rate after removal is about 4% [17].

As mentioned above, mature cystic ovarian teratoma (MCT) can have different histological elements [5, 6]; however, the finding of a well-differentiated cerebellum is extremely rare. The first case reported of a well-differentiated cerebellum within mature teratoma was by Askanazy in 1907 [7]. Furthermore, to the best of our knowledge after an extensive literature review, only 22 cases have been reported [19–22]. The cerebellum formed within ovarian teratoma usually has a less organized morphology [22], and it can be associated with different morphological features such as dendritic abnormalities of Purkinje cells [20] or presence of Obersteiner external granular layer [19].

Well-differentiated cerebellum within ovarian teratoma represents a diagnostic challenge to the pathologist as it shares many similarities with the immature elements in teratoma [22], which has a different prognosis and management modalities depending on the grade and stage [23]. This dilemma can be resolved by identifying that the cerebellar tissue in mature teratoma has more of an organoid architecture versus the haphazardly arranged immature tissue elements seen in immature teratoma [20, 22]. However, this is more elusive and difficult to assess, especially in frozen section/intraoperative consultation, where the external or internal granular layer of the cerebellum can be difficult to differentiate from immature neural tissue [20, 22]

Interestingly, ovarian teratoma with both well-differentiated cerebellum and immature components occurring together has also been reported [24]. So, finding one does not exclude the other. Hereby, we emphasize the importance of extensive sampling of ovarian teratoma and attention to such rare findings.

## Conclusion

We report this case of well-differentiated cerebellum within ovarian teratoma to expand the pool of cases of this extremely rare entity in literature. This finding represents a diagnostic challenge to the pathologist due to its rarity and its similarity to immature teratoma. Furthermore, the presence of both components (immature elements and well-differentiated cerebellum) in ovarian teratoma has been reported in literature. Thus, we emphasize that thorough sampling of ovarian teratoma is of paramount importance and to keep the aforementioned diagnosis in mind and not to confuse it with immature elements, especially in frozen section/intraoperative consultation.

## Data Availability

All the data used in this case report are available from the corresponding author.

## Abbreviations

GCT: Germ cell tumor
H&E: Hematoxylin and eosin
MCT: Mature cystic ovarian teratoma
MOGCT: Malignant ovarian germ cell tumor
SCC: Squamous cell carcinoma
US: Ultrasound
